# Predictive Value of Endometrial Length Measurement by Transvaginal Ultrasound and IVF/ICSI Outcomes

**DOI:** 10.22074/ijfs.2020.44380

**Published:** 2020-10-12

**Authors:** Firoozeh Ahmadi, Amirhossein Maghari, Fattaneh Pahlavan

**Affiliations:** 1 Department of Reproductive Imaging, Reproductive Biomedicine Research Center, Royan Institute for Reproductive Biomedi- cine, ACECR, Tehran, Iran; 2 Atherosclerosis Research Center, Baqiyatallah University of Medical Sciences, Tehran, Iran

**Keywords:** ART, Endometrial Length, Ultrasonography

## Abstract

**Background:**

The purpose of this study to determine the relationship between endometrial length and positive preg- nancy test in patients who underwent assisted reproductive technology (ART).

**Materials and Methods:**

This cross-sectional study included patients who were referred for in vitro fertilisation/in- tracytoplasmic sperm injection (IVF/ICSI) therapy from 2013 to 2016. All nulliparous women who met the inclusion criteria were between 20-38 years of age and presented for ultrasound measurements prior to fresh embryo transfer (ET). Endometrial length was measured by transvaginal ultrasound (TVS) with a Medison Accuvix device on the day of human chorionic gonadotropin (hCG) administration. The relationship between endometrial length and treatment success was assessed. The independent sample t test, receiver operating characteristic (ROC) curve and the area under the curve (AUC) index and chi-square test were used for data analysis. P values <0.05 were statistically significant.

**Results:**

There was a significant relationship between endometrial length (41.5%) and treatment success (P<0.05). The endometrial length of 41.5(mm) with a sensitivity of 66.7%, specificity of 50.6%, positive predictive value of 46.8%, negative predictive value of 69.4%, and efficiency of 56.62% can be used as a proper cut-off point with an AUC of 0.63.

**Conclusion:**

The value of 41.5(mm) for endometrial length can be used as a proper cut-off point for prediction of a higher ART success rate. We recommend TVS as the first step for assessment of uterine and endometrium receptivity in the ART cycle.

## Introduction

Infertility is considered as a global public health issue that affects almost 186 million people worldwide (1). Infertility is a consideration in one out of eight reproductive age women and one out of ten men of reproductive age (2). Despite improvements in assisted reproductive technology (ART), only less than 50% of patients achieve success in terms of live-birth deliveries (3).

A successful pregnancy outcome for patients who undergo ART depends on embryo quality, favourable intrauterine environment, and a skilful* in vitro* fertilisation (IVF) laboratory (4). Another important factor is endometrial receptivity (5, 6).

There are several studies that discuss predictive value of endometrial characteristics in terms of ART success, such as Echo pattern and endometrial thickness (7). However, in women without uterine abnormalities, little is known about uterine and endometrial length (8). In recent studies, a catheter or hysterometer has been used for uterine length measurements. In a similar study, the researchers used transvaginal ultrasound (TVS) and compared implantation and clinical pregnancy rates between groups that had with uterine lengths >7.0 cm, 7-7.9 cm and >9.0 cm. The results were varied and there was much controversy in the findings (9-11).

The question arises as to whether an association exists between the endometrium length (from the internal ostium of the cervix to the uterine fundus) and the incidence of clinical pregnancy in women with normal uterine anatomy who undergo IVF or intracytoplasmic sperm injection (ICSI).

This study aimed to determine the relationship between endometrial length and positive pregnancy in ART patients. We hypothesized that an association exists between uterine length and positive pregnancy test in patients who underwent IVF/ICSI.

## Materials and Methods

### Study population

This cross-sectional study included 166 patients who referred to Royan Institute, Tehran, Iran for IVF/ICSI therapy and ET from 2013 to 2016. The study and protocols were approved by the Royan Institute Ethics Committee (Ethics number: IR.ACECR.ROYAN.REC.1396.146) and informed written consent was obtained from the patients.

All nulliparous women aged 20-38 years of age who presented for ultrasound measurements prior to fresh ET were considered for inclusion. The inclusion criteria consisted of: first IVF/ICSI treatment cycle, absence of any anomaly in the endometrium or myometrium, and no histories of abortion or curettage, hysteroscopy, polypectomy or myomectomy. Patients who had endometrial lengths between 7-14 mm were enrolled. Finally, 166 patients who met the eligibility criteria were selected.

### Uterine length measurements

The OCP-LD cycle was begun for all patients from the second to fifth days of menstruation. From day 17 of menstruation, the patients received daily subcutaneous injections of GnRH to 500 μg/d. At 12-14 days after the GnRH-a, the dose was reduced to 200 μg/d. Then, FSH stimulation was begun according to ovarian reserve and the patient’s age. Subsequently, the dosage was increased or decreased according to the patient's needs.

Assessment of follicle growth and endometrial condition were performed by vaginal ultrasound. If the patient had at least three follicles that were ≥18 mm in two ovaries, we measured the endometrial length (from the internal ostium of the cervix to the fundus) of the uterus by TVS on the day of the human chorionic gonadotropin (hCG) administration (Fig . 1).

**Fig.1:**
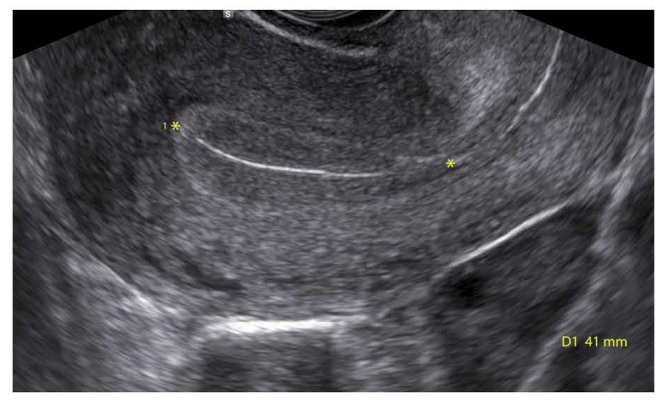
Evaluation of endometrial length of uterus by vaginal ultrasound.

### Outcome measures

Chemical pregnancy was defined as cycles that resulted in the identification of serum beta-hCG but without the subsequent development of a gestational sac. Clinical pregnancy denoted cycles that resulted in ultrasound confirmation of an intrauterine gestational sac. We considered clinical pregnancy as the positive outcome of IVF/ICSI.

### Statistical analysis

Cases were divided into two groups - positive and negative for pregnancy. Variables of age, ET number and endometrial thickness, weight, height and body mass index (BMI) were similar in the positive and negative pregnancy groups.

Data was entered into SPSS Version 21 software for statistical analysis. The relationship between endometrial length and treatment success was assessed. We used the independent sample t test, multiple logistic regression, receiver operating characteristic (ROC) curve and the area under the curve (AUC) index for data analysis. P values<0.05 were considered to be statistically significant.

## Results

Overall, 166 cases (IVF or ICSI) that met the inclusion criteria entered the study. Patients were between 20 and 38 years of age with a mean age of 29.08 ± 4.24 years. The overall pregnancy rate was 39.8%.

The adjusted P value was obtained by adjusting for age, height, weight, BMI, ET and thickness for the relationship between endometrial length and pregnancy (positive and negative). The results showed that weight, height and BMI had a significant effect on this relationship (Table 1).

**Table 1:** Multiple logistic regression results


Variables	B(SE)	P value	OR	95% CI for OR	
				Lower	Upper

Age (Y)	0.002 (0.046)	0.965	1.002	0.916	1.096
Height	-0.763 (0.284)	0.007	0.466	0.267	0.813
Weight(kg)	0.866 (0.338)	0.010	2.379	1.227	4.612
BMI (kg/m^2^)	-2.242 (0.887)	0.012	0.106	0.019	0.605
ET number	-0.361 (0.282)	0.201	0.697	0.401	1.212
Endometrial thickness	0.040 (0.103)	0.698	1.041	0.851	1.273
Endometrial length	-0.094 (0.041)	0.022	0.910	0.839	0.987
Constant	127.632 (46.008)	0.006	26.910	-	-


BMI; Body mass index, ET; Embryo transfer, CI; Confidence interval, OR; Odds Ratio, B; Beta, and SE; Standard error.

The variables of age, ET number, endometrial thickness, weight, height and BMI were similar in the positive and negative pregnancy groups (Table 2).

**Table 2:** Independent t test results for comparing the demographic and laboratory characteristics of the positive and negative pregnancy groups


Variables	Pregnancy				P value^*^
	Positive(N=66)		Negative (N=100)		
	Mean	SD	Mean	SD	

Age (Y)	29.23	3.76	28.98	4.55	0.714
Height(cm)	161.56	5.90	160.25	6.21	0.177
Weight (kg)	66.62	8.49	66.32	11.52	0.847
BMI (kg/m^2^)	25.58	3.42	25.74	3.61	0.772
ET number	2.34	0.55	2.19	0.72	0.167
Endometrial thickness (mm)	11.4	2.1	10.82	2.3	0.08


*; Independent sample t test (statistical significance level: 0.05), BMI; Body mass index, ET; Embryo transfer, and SD; Standard deviation.

The endometrial length range from 28-58 mm and thickness range was 7-14 mm. We initially used the ROC curve and AUC index to determine the cut-off length of the endometrium in terms of treatment success and positive pregnancy (Fig . 2).

**Fig.2:**
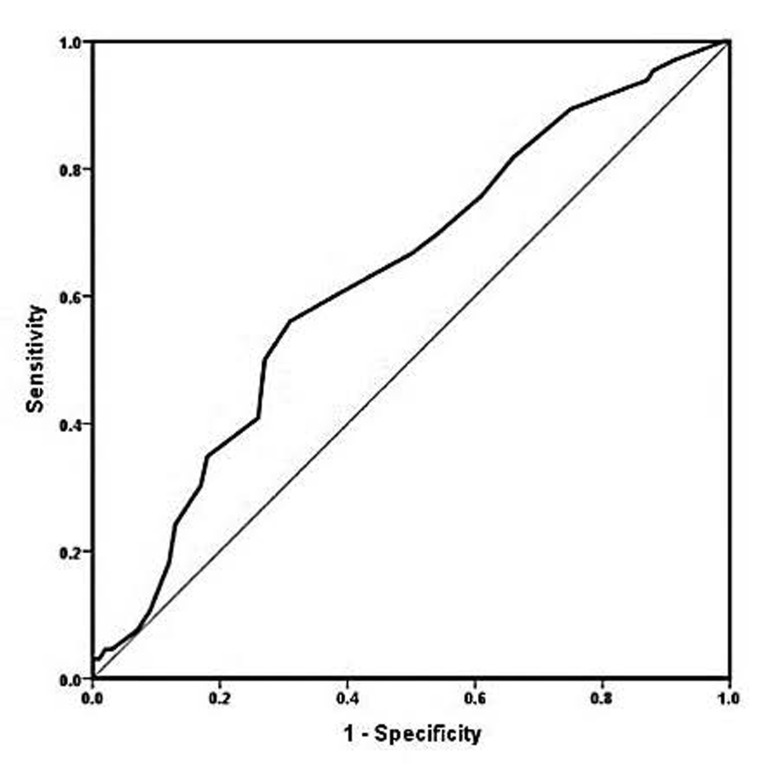
Receiver operating characteristic (ROC) curve.

We determined that the AUC was 0.63, which was acceptable and significant because it was greater than 0.6 (P<0.05, Fig . 2, Table 3). Thus, the cut-off point was determined to be 41.50, which could be used as a proper cut-off point.

**Table 3:** AUC index results


AUC	SE	P value	95% CI	
			Lower bound	Upper bound

0.630	0.044	0.0044	0.544	0.716


AUC; Area under the curve, CI; Confidence interval, and SE; Standard error.

There was a significant relationship between endometrial length and treatment success (P<0.05, Table 4).

**Table 4:** Chi-square test results for new cut of point


Endometrium length		Pregnancy		Total	P value
		Positive(n=66)	Negative(n=100)		

≤ 41.5	N	22	50	72	0.034
	Endometrium length (%)	30.6%	69.4%	100%	
	Pregnancy rate (%)	33.3%	50%	43.4%	
> 41.5	N	44	50	94	
	Endometrium length (%)	46.8%	53.2%	100%	
	Pregnancy rate (%)	66.7%	50%	56.6%	


We determined that the 41.5 value had a sensitivity of 66.7%, specificity of 50.6%, positive predictive value of 46.8%, negative predictive value of 69.4%, and efficiency of 56.62% and could be used as a proper cut-off point.

## Discussion

Infertility treatments are expensive. Because the implantation rates are low, it is necessary to find a way to predict the success of an ART cycle (12).

There are limited studies about the diagnostic value of endometrium length using TVS in infertile women who undergo IVF/ICSI and the determination of its cut-off. We found that the IVF/ICSI success was higher in cases that had greater endometrial length. In this study, we noted that the value of 41.5 with a sensitivity of 66.7% and specificity of 50.6% could be used as a proper cut-off point with an AUC of 0.63. This was the first time that an endometrial length cut-off point for IVF/ICSI has been assessed.

A similar study that assessed the predictive value of endometrial length and success of the IVF/ICSI confirmed our result. Abdel et al. stated that the depth of ET is one of the most important factors in IVF/ICSI adaption (13). An appropriate endometrial length is necessary. In the current study, we concluded that IVF/ICSI success was higher in cases that had higher endometrial lengths.

As a physiological view, we noted the effects of oestrogen on the endometrium and success of the ART cycle, which correlated with uterine size (9). Increased endometrial length observed by TVS might be an index for a sufficient hormonal level and appropriate environment of uterine, and would result in a better ART outcome.

Hawkins et al. measured the uterine length (from the fundus to the external ostium of the cervix) in ART cycles before ET. They noted that the implantation rate and clinical pregnancies were higher in cases with uterine lengths between 7 and 9 cm, which were consistent with our finding, although it was not statistically significant (8). In contrast, Firouzabady et al. did not report any association between uterine length and IVF/ICSI adaption (10, 11).

There were some differences between our study and previous studies. We measured the endometrial length with TVS; however, we omitted the cervix length from the measurement. Therefore, our assessment and its relationship with ART outcome would be more logical. The endometrial length was measured by an experienced sonographer in order to eliminate any inter-observer bias.

Some studies confirmed the positive effect of endometrial thickness in success of IV. For this, we math cases in term of endometrial thickness. Momeni conducted a meta-analysis and reported that women, who underwent IVF which resulted in positive pregnancy outcomes, had higher mean endometrial thicknesses compared with a non-pregnant group (14).

We suggest that additional studies be conducted with larger sample sizes. The combined uterine index cut-off points and profile for ART success that includes thickness, echo pattern, position and length might improve the ART outcome. More studies should evaluate these findings.

## Conclusion

We determined that the value of 41.5 for endometrial length had appropriate sensitivity, specificity, positive predictive value, negative predictive value and efficiency, and could be used as a proper cut-off point for prediction of a higher ART success rate. We recommend TVS should be performed as the first step for uterine and endometrium receptivity assessment in the ART cycle.
